# Immunostimulatory responses to crude extracts of *Warburgia ugandensis* (sprague) subsp *ugandensis* (canellaceae) by Balb/c mice infected with *Leishmania major*

**DOI:** 10.11694/pamj.supp.2014.17.1.3638

**Published:** 2014-01-18

**Authors:** Peter Ngure, Zipporah Ng'ang'a, Albert Kimutai, Stella Kepha, Samuel Mong'are, Johnnie Ingonga, Willy Tonui

**Affiliations:** 1 Daystar University, Kenya; 2 Jomo Kenyatta University of Agriculture and Technology; 3 Kabianga University, Kenya; 4 Kenya Medical Training College, Kenya; 5 Kenya Medical Research Institute, Kenya

**Keywords:** Leishmaniasis, immunostimulatory responses, *Warburgia ugandensis*, cytokines

## Abstract

**Introduction:**

To determine the immunostimulatory potential of crude extracts of *Warburgia ugandensis* subsp. *ugandensis* with a soluble leishmanial antigen in vaccinating BALB/c mice.

**Methods:**

Seventy two female BALB/c mice were randomly assigned into six groups. The mice were vaccinated with soluble *leishmania* antigens (SLA) alone, hexane, ethyl acetate, and dichloromethane extract co-administered with SLA. Unvaccinated mice formed the control group. The induction of cell-mediated immunity following vaccination was determined by measuring in vitro lymphocyte proliferation and the production of interleukin (IL)-4 and gamma interferon (IFN-γ) determined by flow cytometry. Protection against L. major was determined by quantifying parasite burdens in *L. major* infected footpads using a limiting dilution assay and by measuring lesion sizes of the infected footpad compared to the contralateral uninfected footpad.

**Results:**

On vaccination with extracts of *W. ugandensis* subsp. *ugandensis* alone or as adjuvants when used in combination with *Leishmania* antigens, the hexane extract and the dichloromethane extract plus SLA stimulated moderate production of IFN-γ and low levels of IL-4.These mice were partially protected from cutaneous leishmaniasis as shown by the slow development of lesions and comparatively less parasite burdens.

**Conclusion:**

These data suggest that extracts of *W. ugandensis* subsp. *ugandensis* are suitable adjuvants for *Leishmania* vaccines. However, since *W. ugandensis* subsp. *ugandensis* has been shown to be effective against *Leishmania* parasites in vitro and in vivo, further studies ought to be conducted to determine its immunochemotherapeutic potential when co-administered with a soluble leishmanial antigen in vaccinating BALB/c mice.

## Introduction

Leishmaniasis is a parasitic disease caused by protozoa of the genus *Leishmania* transmitted by the bite of infected phlebotomine sand flies. It encompasses a wide spectrum of clinical manifestations ranging from chronic cutaneous ulcers to fatal visceral infection. The World Health Organization estimates that at least 350 million people are at risk of the disease worldwide, 12 million people are infected with *Leishmania* parasites, and two million new cases of leishmaniasis occur each year in 88 countries in parts of Africa, India, the Middle East, southern Europe, and Central and South America [1].

The primary challenge associated with the treatment of leishmaniasis is that the drugs of choice are highly toxic, expensive and require hospitalization for more than three weeks. They include pentostam, pentamidine, miltefosine, and paromomycin. There are efforts towards the development of drugs based on natural products which are deemed to be safe. In this regard, medicinal plants offer prospects for discovering new compounds with therapeutic properties.

*Warburgia ugandensis* subsp *ugandensis* Sprague, the East African greenheart(Canellaceae) is one of the most highly utilized medicinal plants in tropical and subtropical Africa and is now highly endangered in the wild [2–3]The dried bark of the tree is commonly chewed and the juice swallowed as a remedy for stomach ache, constipation, toothache, venereal diseases, cough, fever, muscle pains, weak joints and general body pains. The leaf decoction baths are used as a cure for skin diseases while the bark, roots or leaves can be boiled in water and drunk to treat malaria, although this causes violent vomiting. *Warburgia ugandensis* subsp *ugandensis* which is known as “soket” in Tugen tribe is used by traditional healers to treat VL (W. Tonui, personal communication). The stem barks are taken orally in boiled water or soup. Previous studies on *W. ugandensis* subsp ugandensis have shown good antibacterial, antifungal, antiviral activity and trypanocidal effects. For example, crude extracts and purified compounds of *W. ugandensis* subsp *ugandensis* showed activity against Mycobacterium tuberculosis H37Rv and M. Bovis BCG Pasteur [4], Candida albicans and measles virus [5]. Similarly, the activity of *W. ugandensis* subsp *ugandensis* against trypanosomes has been demonstrated [5–6]. However, there is limited information on the immunostimulatory effects of the plant. The objective of the present study was to determine the immunostimulatory effects of extracts of *W. ugandensis* subsp *ugandensis* against *Leishmania major* and *L. donovani* in BALB/c.

## Methods

### Collection of *W. Ugandensis* subsp. *ugandensis* plant materials

*Warburgia ugandensis* subsp *ugandensis* stem barks were collected from Sesia village in Kabarnet, Baringo District, in the Rift Valley Province in Kenya. Sesia village is located 265 km northwest of Nairobi on the Tugen Hills on the western edge of the Rift Valley (Grid Reference 056 547 on the 1:50,000 sheet 104/1 Kipkabus).Botanical identification was carried out by botanists from the National Museum of Kenya.

### Preparation and extraction of *W. Ugandensis* subsp *ugandensis* extracts

The stem barks were cut into small pieces and air-dried for three weeks. The dried specimens were shred using an electrical mill in readiness for extraction.The sample preparation and extraction was carried out as described by Harborne [7]. Successive extraction was carried out on the plant material with distilled organic solvents of increasing polarity, which included n-hexane, dichloromethane, ethyl acetate and methanol. Three hundred grams of the shred stem barks was weighed and put in 1 litre conical flasks. Six hundred millilitres of n-hexane was added and soaked for 48 hours. The residue was filtered using a Buchner funnel under vacuum until the sample dried.The sample was soaked further with 600ml of n-hexane for 24 hours until the filtrate remained clear.The filtrate was then concentrated under vacuum by rotary evaporation at 30-35°C to yield a brown residue^7^.The brown residue was transferred to a sample bottle and dried under vacuum; the weight of the dry extract was recorded and stored at -20°C until required for bioassay. The process was repeated sequentially for dichloromethane, ethyl acetate and methanol.

### Bioassays

Six week-old BALB/c mice were obtained from KEMRI'S animal house facility and used for the in vivo studies. These experiments were carried out in compliance with all relevant KEMRI institutional policies.

### *In Vitro* parasite culture for *L. Major*

Metacyclicpromastigotes of *L. Major* strain (Strain IDU/KE/83 =NLB-144) and *L. Donovani* strain (NLB-065) were used. Parasites were maintained as previously described by Titus et al. 1984 [8] and metacyclics were isolated from stationary phase cultures by negative selection using peanut agglutinin [9].Stationary-phase promastigotes were obtained from 5 to 7-day-old cultures. The cultures were made in T25 sterile disposable culture flasks (25 ml) and incubated at 25°C as recommended by Evans et al. 1989 [10] to stationary metacyclic stage.

### Preparation of extracts for bioassays

Stock solutions of the extracts were made in culture media for anti-leishmanial assay and re-sterilized by passing through 0.22 µm micro-filters under sterile conditions in a laminar flow hood.The extracts that were insoluble in water or media were first dissolved in 1% DMSO to avoid solvent carry over [11].All the extracts were stored at 4°C until use.

### Preparation of soluble *Leishmania* antigens

Soluble *Leishmania* antigens (SLA) were produced as previously described by Scott and colleagues [12]. Promastigotes (1 × 109) were harvested from culture and washed four times in cold phosphate-buffered saline (PBS) and resuspended in PBS containing a protease inhibitors cocktail of 100 Mm Tris-HC1, 1m EDTA (pH 8) supplemented with 50 µg/ml leupeptin, 50µg/ml antipapain, 50 µg/ml aprotinin and 1.6 mMphenylmethylsulfonyl fluoride (PMSF) (Sigma Chemicals Co.). The suspension was incubated for 10 min on ice and sonicated for 10 periods of 30 seconds each (separated by an interval of 1 min) at medium amplitude. The sonicate was left at 4°C for complete extraction of soluble antigen for 18 h. After incubation, sonicated suspension was centrifuged at 4000g for 30 min at 4°C. The supernatant obtained was finally ultra-centrifuged at 40000 g for half an hour. After assessing the protein content of the supernatant by Lowry method, it was distributed in small aliquots and stored at -70°C until use^13^.

### Infection of mice, treatment and determination of parasite numbers in cutaneous lesions

Female BALB/c mice were infected in the hind footpad with 1 × 106 *L. major* metacyclicpromastigotes. In all experiments, treatment was initiated 4 or 5 weeks after infection had established as determined by the presence of lesions [12]. Lesion development was followed by measuring the thickness of the infected footpad as compared to the thickness of the same footpad prior to infection using a verniercaliper. Disease progression in mice was evaluated by measuring lesion sizes for a period of eight weeks on a week-to-week basis. At 1, 2 and 4-weeks post infection, duplicate mice were sacrificed to estimate the parasite burden in the footpads using a limiting dilution assay (LDA)[14]. Mice that were not vaccinated formed the control groups.

The extracts were injected intraperitoneally. The untreated group received Phosphate buffered saline. Lesion development was followed by measuring thickness of the infected footpad as compared to the thickness of the contralateral footpad prior to infection using a vernier caliper. All mice were sacrificed during the 12th week, spleens weighed and impression smears made. These were fixed in absolute methanol and stained with Giemsa before examination for parasites to determine whether or not visceralization of the parasite had taken place.

### Immunostimulatory effects of crude extracts derived from *W. Ugandensis* subsp. *Ugandensis*

To test the immunostimulatory effects of the crude extracts, soluble *Leishmania* antigens (SLA) were used. Female BALB/c mice in groups of ten were vaccinated by subcutaneous (s.c) injections of 100µg of SLA alone, 100µg of test sample alone or with 100µg of SLA plus 100µg of test sample [9].

### Cell cultures, proliferation of cytokine producing cells and measurement of cytokines

Spleens from mice infected with *L. Major* were harvested in each experimental group on day 0 and at days 7, and 21 post-infection. Mononuclear cells were purified with Ficoll-Paque ingredients [9]. Spleen cells were adjusted to 106/ml in complete RPMI 1640 medium supplemented with 10% fetal bovine serum (FBS), 2 mM L-glutamine, 100 units of penicillin per ml, and 100 µg of streptomycin per ml then stimulated with SLA for six days at 37°C and 5% CO2.Negative control cultures consisted of unstimulated splenocytes. Viability of splenocytes was tested by stimulating the cells with a known mitogen, concanavalin A (con A). Colorimetric microassay was used to determine cell proliferationas previously described by Hay and Westwood [15]. The cytometric bead array (CBA) kit was used to measure levels of IFN-γ and IL-4 as previously described by Hodge et al.[16].

### Ethical and biosafety considerations

Approval for the study was sought from Kenya Medical Research Institute (KEMRI) ethical review committee. The experiments were done in compliance with KEMRI's Animal Care and Use Committee (ACUC).

### Statistical analysis

Data were recorded in Microsoft Excel and imported into SPSS 13.0 for analysis. All experiments were carried out in triplicate. The mean and standard deviation of at least three experiments were determined. Statistical analysis of the differences between mean values obtained for the experimental groups were done by Student's t test. P values of ≤0.05 or less were considered significant.

## Results

### Lymphoproliferative responses of splenocytecultures beforeand after infection with *L. major*

The proliferative responses of splenocyte cultures to SLA obtained from mice that had been vaccinated with SLA, hexane, dichloromethane and ethyl acetatewas significantly higher (P<0.05) to the unvaccinated control (Table 1). Combination of SLA and extracts yielded similar results.There was marked increase in the proliferative response to SLA for the groups vaccinated with SLA, hexane extractand dichloromethane extractplus SLA compared to the proliferation before infection (P<0.05).

**Table 1:** Mean optiocal density (OD) readings (540 nm) of in vitro proliferative responses of splenocytes one week and four weeks post vaccination

Treatment groups	Proliferative responses one week after vaccination			Proliferative responses four weeks after vaccination		
	Mean OD readings ( 540 nm) ± S.E			Mean OD readings (540 nm) ± S.E		
	LA	Medium	P value compared to the unvaccinated control	SLA	Medium	P value compared to the unvaccinated control
SLA	0.636 ± 0.02	0.355± 0.037	0.002	1.069± 0.072	0.378 ± 0.007	< 0.001
HEX	0.540± 0.013	0.335 ± 0.07	0.002	0.953± 0.004	0.365 ± 0.05	0.118
DCM	0.511± 0.028	0.393± 0.007	0.009	0.597± 0.023	0.403 ± 0.011	0.002
ETAC	0.536± 0.003	0.258± 0.003	< 0.001	0.598± 0.024	0.298 ± 0.008	0.023
HEX/SLA	0.737± 0.027	0.278± 0.007	0.004	0.328± 0.024	0.279 ± 0.008	0.02
ETAC/SLA	0.868± 0.067	0.340± 0.011	0.014	0.304± 0.004	0.347 ± 0.002	0.006
DCM/SLA	0.587± 0.010	0.289± 0.014	0.002	0.746± 0.009	0.307 ± 0.09	0.042
PBS	0.268± 0.005	0.230± 0.003	-	0.268± 0.005	0.256 ± 0.006	-

SLA=Soluble *Leishmania* antigen; HEX=Hexane; DCM=Dichloromethane; ETAC=Ethyl acetate; HEX/SLA=Hexane/SLA; ETAC/SLA=Ethyl acetate/SLA; DCM/SLA=Dichloromethane/SLA; PBS=Phosphate Buffered Saline

### Interferon gamma production by splenocyte cultures before and after infection with *L. major*

The mean production of IFN-γ by splenocytes of mice vaccinated with SLA, hexane extract, dichloromethane extract, ethyl acetate, hexane plus SLA, ethyl acetate plus SLA, and dichloromethane plus SLA in response to in vitro stimulation with SLA before challenge was significantly higher (P<0.05) compared to the control unvaccinated group (Table 2). The mean production of IFN-γ by splenocytes of mice vaccinated withSLA, hexane extract, dichloromethane extract, ethyl acetate, hexane plus SLA, ethyl acetate plus SLA, and dichloromethane plus SLA,in response to in vitro stimulation with SLA after challenge was significantly higher (P<0.05) compared to the control unvaccinated group.

**Table 2:** In vitro IFN-γ production (mean ± SE) one week and four weeks after vaccination

Treatment groups	Mean production of IFN-γ one week after vaccination			Mean IFN-γ production four weeks after vaccination		
	Mean OD readings ( 540 nm) ± S.E			Mean OD readings (540 nm) ± S.E		
	Mean Production of IFN-γ (pg/ml) induced by SLA	Mean production of IFN-γ (pg/ml) by cells in medium	P value compared to the unvaccinated control	Mean production of IFN-γ (pg/ml) induced by SLA	Mean production of IFN-γ (pg/ml) by cells in medium	P value compared to the unvaccinated control
SLA	349.1± 9.825	6.0 ± 1.971	0.002	123.2± 2.730	5.6 ± 0.345	0.002
HEX	134.3± 7.549	7.07±0.368	0.005	141.8± 1.205	6.7 ± 0.211	0.005
DCM	142.0± 3.132	7.8 ± 0.988	0.006	142.9± 5.775	7.0 ± 0.548	0.032
ETAC	116.1± 2.119	5.6 ± 0.731	<0.001	112.9± 2.905	6.4 ± 0.706	< 0.001
HEX/SLA	111.2± 4.932	6.7 ± 0.575	0.028	135.4± 5.084	6.1 ± 1.977	0.012
ETAC/SLA	138.2± 1.376	5.8 ± 0.309	< 0.001	109.2± 2.960	6.3 ± 0.367	0.017
DCM/SLA	167.3± 4.350	6.2 ± 0.889	0.005	120.6± .684	5.8 ± 0.506	0.005
PBS	65.8± 2.892	5.3 ± 0.070	-	63.5 ± 6.612	5.7 ± 0.172	-

SLA=Soluble *Leishmania* antigen; HEX=Hexane; DCM=Dichloromethane; ETAC=Ethyl acetate; HEX/SLA=Hexane/SLA; ETAC/SLA=Ethyl acetate/SLA; DCM/SLA=Dichloromethane/SLA; PBS=Phosphate Buffered Saline

### Interleukin - 4 production by splenocytecultures before and after infection with *L. major*

The mean production of IL-4 by splenocytes of mice vaccinated with SLA, hexane extract, dichloromethane extract, ethyl acetate, ethyl acetate plus SLA, and dichloromethane plus SLA)in response to in vitro stimulation with SLA after challenge was significantly higher (P<0.05) compared to the control unvaccinated group. The mean production of IL-4by splenocytes of mice vaccinated with hexane extract, dichloromethane extract, ethyl acetateand dichloromethane plus SLA in response to in vitro stimulation with SLA after challenge was significantly lower (P<0.05) compared to the control unvaccinated group.

The production of IL-4 by splenocytes from mice vaccinated with hexane plus SLA was not significantly different (P=0.245) from that of the unvaccinated control (Table 3), while the levels of production of IL-4 for mice vaccinated with ethyl acetate extract plus SLA and SLA, were significantly higher (P< 0.001) compared to that of the unvaccinated control.

**Table 3:** In vitro IL-4 production (mean ± SE) one week and four weeks after vaccination

Treatment groups	Mean production of IL-4 one week after vaccination			Mean IL-4 production four weeks after vaccination		
	Mean OD readings ( 540 nm) ± S.E			Mean OD readings (540 nm) ± S.E		
	Mean Production of IL-4 (pg/ml) induced by SLA	Mean production of IL-4 (pg/ml) by cells in medium	P value compared to the unvaccinated control	Mean production of IL-4 (pg/ml) induced by SLA	Mean production of IL-4 (pg/ml) by cells in medium	P value compared to the unvaccinated control
SLA	39.3 ± 2.563	6.2 ± 0.642	0.002	71.6 ± 0.329	7.5 ± 0.384	< 0.001
HEX	33.9 ± 2.237	6.4 ± 0.656	0.005	36.9 ± 0.525	7.1 ± 0.379	0.001
DCM	34.8 ± 3.303	7.3 ± 0.464	0.004	32.1 ± 0.414	6.9 ± 0.312	< 0.001
ETAC	29.5 ± 1.424	7.4 ± 0.327	0.022	31.2 ± 0.476	7.4 ± 0.658	< 0.001
HEX/SLA	22.9 ± 0.983	7.9 ± 0.543	0.245	65.4 ± 1.762	7.4 ± 0.312	0.915
ETAC/SLA	33.4 ± 1.688	7.7 ± 0.719	0.003	74.0 ± 0.732	7.3 ± 0.478	< 0.001
DCM/SLA	36.3 ± 1.227	7.8 ± 0.503	0.036	32.9 ± 0.492	8.4 ± 0.251	< 0.001
PBS	21.1 ± 2.115	6.7 ± 0.165	-	65.7 ± 0.541	6.7 ± 0.175	-

SLA=Soluble Leishmania antigen; HEX=Hexane; DCM=Dichloromethane; ETAC=Ethyl acetate; HEX/SLA=Hexane/SLA; ETAC/SLA=Ethyl acetate/SLA; DCM/SLA=Dichloromethane/SLA; PBS=Phosphate Buffered Saline

### Lesion size measurements for BALB/c mice vaccinated before infection with *L. major*

For mice vaccinated with hexane extract, the size of lesions were significantly smaller (P<0.05) than those of the unvaccinated controls from week 2 to week 8. For mice vaccinated with dichloromethane extract, ethyl extract, hexane extract plus SLA and ethyl acetate extract plus SLA there was no significant difference (P>0.05) between the lesion sizes of these mice compared to those of the unvaccinated controls from week 2 to week 8 (Table 4).

**Table 4:** Parasite burden in the infected footpads of mice treated with BCG, SLA, hexane extract, dichloromethane extract, ethyl acetate extract and control (PBS) group alone during the course of infection

	Experimental groups (No. of *L. major* (10^6^)/footpad (95% confidence limits)				
Day	Control	SLA	Hexane extract	Dichloromethane extract	Ethyl acetate extract
14	10.5 (28.9-2.67)	11.4 (18.31-3.16)	6.99 (13.6-1.71)	12.6 (17.8-9.61)	14.3 (16.1-5.28)
35	20.4 (37.35-13.83)	18.4 (27.7-12.25)	8.27 (21.01-3.38)*	25.8 (31.74-17.2)	23.6 (32.78-16.9)
56	69.8 (98.1-38.44)	52.3 (72.37-19.4)*	9.33 (7.20-0.93)*	53.5 (82.5-21.37)*	48.8 (62.1-39.04)

* *P*<0.05 Parasite burden is significantly different from that of the unvaccinated control.

Mice in the control unvaccinated and *L. major* challenged group developed large progressive footpad lesions reaching a peak at week (mean ± S.E., 0.947 ± 0.022) mm. Unvaccinated control groups were euthanatized if they reached a predetermined level of lesion severity and/or displayed clinical signs of distress or pain.

### Quantification of *L. major* in infected footpads of BALB/c mice vaccinated before and after infection with *L. major*

The number of *L. major* parasites present in the infected footpads was quantified at 14, 35 and 56 days post infection using a limiting dilution assay (LDA) [15]. The results were analyzed using the ELIDA statistical package (2001-2005). In the first LDA done at 14 days post infection, *L. major* parasites were detected in both the vaccinated and unvaccinated groups of mice. The number of *L. major* in the footpads from mice vaccinated with dichloromethane extract plus SLA (9.25 &time; 106) was significantly lower (P<0.05) than that of the controls (10.5 × 106) at 14 days post infection. However, there was no significant difference (P<0.05) in the number of parasites for the rest of the groups compared to the unvaccinated control (Table 5).

**Table 5:** Parasite burden in the infected footpads of mice treated with hexane/SLA, dichloromethane/SLA, ethyl acetate/SLA and control (PBS) group alone during the course of infection

	Experimental groups (No. of *L. major* (10^6^)/footpad (95% confidence limits)			
Day	Control	Hexane/SLA extract	Ethyl acetate/ SLA extract	Dichloromethane/SLA extract
14	10.5(28.9-2.67)	9.25(24.7-3.39)	11.7(19.62-6.94)	9.25(10.7-1.12)*
35	20.4(37.35-13.83)	19.03(31.62-14.56)	20.97(26.8-15.72)	8.11(10.16-1.37)*
56	69.8(98.1-38.44)	48.83(87.21-29.73)*	63.7(88.9-38.7)	6.78(7.39-0.96)*

* *P*<0.05 Parasite burden is significantly different from that of the unvaccinated control.

The parasite burdens in the mice vaccinated with hexane extract (8.27 x 106) and dichloromethane extract plus SLA (8.11 × 106) were significantly lower (P< 0.05) from those of the control unvaccinated mice as the disease progressed as quantified at day 35. On the 14th day, there was no significant difference (P<0.05) in the number of parasites for the rest of the groups compared to the unvaccinated control (Table 5).

By the 56th day, parasite burdens for themice vaccinated with hexane extract group (9.33 × 106) and the dichloromethane plus SLA group (6.78 × 106) reduced significantly (P<0.005) compared to those of the unvaccinated control group (69.8 × 106). Similarly, parasite burdens for the SLA group (52.3 × 106), dichloromethane group (53.5 × 106), ethyl acetate group (48.8 × 106), hexane plus SLA group (48.8 × 106) and ethyl acetate plus SLA group (63.7 × 106) reduced significantly (P<0.05) compared to the unvaccinated controls (69.8 × 106). The parasite burdens of the dichloromethane, and the dichloromethane plus SLA were significantly lower (P< 0.005) compared to thoseof the SLA group, dichloromethane group, ethyl acetate group, hexane plus SLA group and ethyl acetate plus SLA group.

## Discussion

The study focused on the immunostimulatory activities of the crude extracts of a native medicinal plant used by traditional healers in Kenya. The limitation of the study is that the active principles responsible for the immunostimulatory responses observed were not identified. Further, the mechanisms of the immunostimulatory activities were not elucidated.

Our results show that organic extracts of *W. ugandensis* subsp *ugandensis* can induce lymphocyte proliferation to SLA on their own or enhance such response if co-administered with SLA in a vaccine.Lymphocyte proliferation is a crucial event in the activation of both cellular and humoral responses^17^. Proliferative responses of splenocytes to SLA have previously been used as a measure of exposure to the parasites, as well as a measure of protection [18].Our resultsare consistent with previous studiesthat suggest that some plant products, such as artemisinin, induce proliferation of T-cells in vitro [19]. The enhanced proliferative responses for extracts co-administered with SLA in this study is consistent with findings of a previous study where mice vaccinated with SLA and Th1 promoting adjuvant had significantly higher stimulation indices compared to those vaccinated with SLA alone^18^. Thirty days post infection with *L. major* there was marked increase in the proliferative response to SLA for the groups vaccinated with SLA, hexane extract and dichloromethane extract plus SLA compared to the proliferation before infection indicating a recall proliferative response. For mice vaccinated with hexane extract plus SLA and the group vaccinated with ethyl acetate plus SLA, the proliferative responses of the splenocyte cultures to SLA was significantly lower. The reduction in the proliferative responses is probably an indication of immunosuppression caused by the combination of SLA with the plant extracts.

Extensive studies with experimental mouse models infected with *L. major* have shown that the outcome of infection with *Leishmania* parasites is critically dependent on the activation of one of the two subsets of CD4 T cells, Th1 and Th2 [20, 21]. In the present study, IFN-γ was expressed in splenocytes, which is in agreement with the findings of other authors who have shown that this cytokine is important in the development of protective immune responses in experimental Leishmaniasis^22^, ^23^]. Gamma interferon secreted by Th1 cells, is the most potent macrophage-activating cytokine leading to host resistance to infection with *Leishmania* parasites whereas IL-4 secreted by Th2 cells, is associated with down-modulation of IFN-γ -mediated macrophage activation[21].

In a previous study, 67 tannins were tested for antileishmanial and immunomodulatory potencies and shown to augment and prolong the activation of host defense mechanisms indicating that parasitized macrophages were “primed” to react to activating molecules such as phenol [24]. Further studies demonstrated the effects of IFN-γ, lipopolysaccharide (LPS), and some polyphenols as individual stimuli, as well as in various combinations on NO production in non-infected and infected macrophage-like RAW 264.7 with emphasis on the NO/parasite kill relationship. In non-infected and in *Leishmania* parasitized cells, gallic acid significantly inhibited the IFN-γ and LPS-induced NO detected in the supernatants [25]. This effect was less prominent in IFN-γ - than in LPS-stimulated cells.

Elsewhere, it has been shown that the ethyl acetate portion of Geranium pyrenaicum induced significant production of IFN-γ and TN [26]. The cytokine-inducing potential of the flavonoids glycosides decreased with increasing polarity [26]. The results provide evidence that flavonoids glycosides possess the capability to stimulate defense mechanisms in *leishmania*-infected RAW 264.7 cells, albeit with moderate potential. This is similar to the observation in the current study where the hexane extract (with low polarity) stimulated production of higher levels of IFN-γ compared to the ethyl acetate extract (higher polarity).

In experimental CL, it has been suggested that the healing of lesions depends on the early presence of macrophages, the activation of these cells by type 1 interferon and, later, by low amounts of IFN-γ probably produced by NK cells, consequently, the Th1 cells amplify the killing of the parasite when they release more IFN-γ [27]. Interferon-gamma is known to be a strong inducer of the macrophage enzyme inducible nitric oxide synthase (iNOS)[28]. which mediates the production of nitric oxide that directly kills *L. major*[29].

Previous studies have demonstrated that CL is associated with the expansion of CD4+ T cells that contain high levels of IL-4 mRNA in the draining lymph nodes and spleen[30] and with elevated levels of immunoglobulin E, an IL-4-dependent immunoglobulin isotype [30].Early production of IL-4 in mice correlates with the dominance of an IL-4-driven Th2 response that causes disease[21]. In the current study the parasite burdens for the SLA, hexane extract plus SLA and ethyl acetate plus SLA groups were comparable to those of the unvaccinated control group four weeks post infection.Indeed by the end of week eight, the lesion sizes of the mice in these groups were similar in size to those of the unvaccinated group indicating exacerbation of disease.

It is postulated that production of high levels of IL-4 interferes with the production or activity of IFN-γ. A number of mechanisms by which IL-4 may interfere with the production or activity of IFN-γ has been identified, including the direct inhibition of macrophage activation to kill intracellular amastigotes[31]. The ability of IL-4 to block the activation of macrophages has been most consistently demonstrated following prior treatment with IL-4. Indeed, administration of IL-4 after IFN-γ may activate macrophage anti-*L. major* activity [32].

These mice were partially protected from cutaneous leishmaniasis as shown by the slow development of lesions. Similar results were reported in immunized BALB/c mice with KM+lectin of Artocarpusintegrifolia associated, or not with SLA, and partial protection of the animals was observed after challenge with L. amazonensis [22].

Mice vaccinated with SLA, ethyl acetate extract, dichloromethane extract and the ethyl acetate plus SLA developed lesions and had higher parasitaemia comparable to the unvaccinated control group. This is in agreement with previous studies where immunization with SLA alone resulted in lesions almost as evident in the controls immunized with PBS [33, 34].

## Conclusion

These results suggested that extracts from *W. ugandensis* subsp *ugandensis* contained chemical compounds that possess positive and/or negative modulator effects on the immune system. *Warburgia ugandensis* subsp. *ugandensis* has been used by traditional healers in Kenya for many years. The immunostimulatory effects of the plant can be used to explain the efficacy of this plant in addition to its direct effects on *Leishmania* parasites. Further studies on the mechanisms have potential of unveiling the possibility of targeting this plant for drug development that can be used for the treatment of leishmaniasis.
